# P01.44. *In vivo* immune modulating effects of Ashwagandha (Withania somnifera)

**DOI:** 10.1186/1472-6882-12-S1-P44

**Published:** 2012-06-12

**Authors:** A Erlandsen, J Mikolai, A Murison, S Mukherjee, H Zwickey

**Affiliations:** 1 Helfgott Research Institute, National College of Natural Medicine, Portland, USA

## Purpose

The herb Ashwagandha (Withania somnifera, Dunal), has been used for centuries in Ayurveda as rasayana – promoter of longevity, well-being, and disease prevention. Traditional use combines the herb with a carrier substance called “anupana,” believed to help aid in bioavailability and absorption. To compare the effects of Ashwagandha root extract plus anupana on human immune activation to Ashwagandha extract without anupana, anupana plus sham extract, and sham extract only.

## Methods

Twenty participants were divided into four groups: Group 1 Ashwagandha root extract only; Group 2 Ashwagandha root extract plus anupanal; Group 3 sham extract plus anupana; and Group 4 sham extract only. Participants took 3mL of their assigned extract twice daily for five days. Organic cow’s milk was used as anupana; Groups 2 & 3 consumed 8 fl.oz. of milk with each dose of extract. Sham extract was 45% grain alcohol: 55% spring water, analogous to the Ashwagandha extract solvent. Four types of lymphocytes were isolated from peripheral blood samples taken at baseline, 24 hours, and 96 hours and compared for differences in surface receptor expression of CD4, CD8, CD19, CD56, and CD69. Data were collected using FACScan flow cytometry. Statistical analyses were completed using ANOVA to investigate between-group effects and by student t-test to investigate within-group effects.

## Results

ANOVA revealed no significant between-group effects. No significant within-group effects were seen in Groups 1, 3, or 4. Within-group analysis of Group 2 from baseline to final demonstrated statistically significant increases in CD69 expression (p<0.009) and an increase in the absolute number (p<0.05) of CD8 T cells. There was also a trend toward increased activation of CD4 T cells after 24 hours.

## Conclusion

Possible clinical implications of these results include prophylaxis and treatment of infectious agents (specifically intracellular parasites and viruses), adjuvant therapy against tumor cells, and effects on hypersensitivity and inappropriate immune balance.

